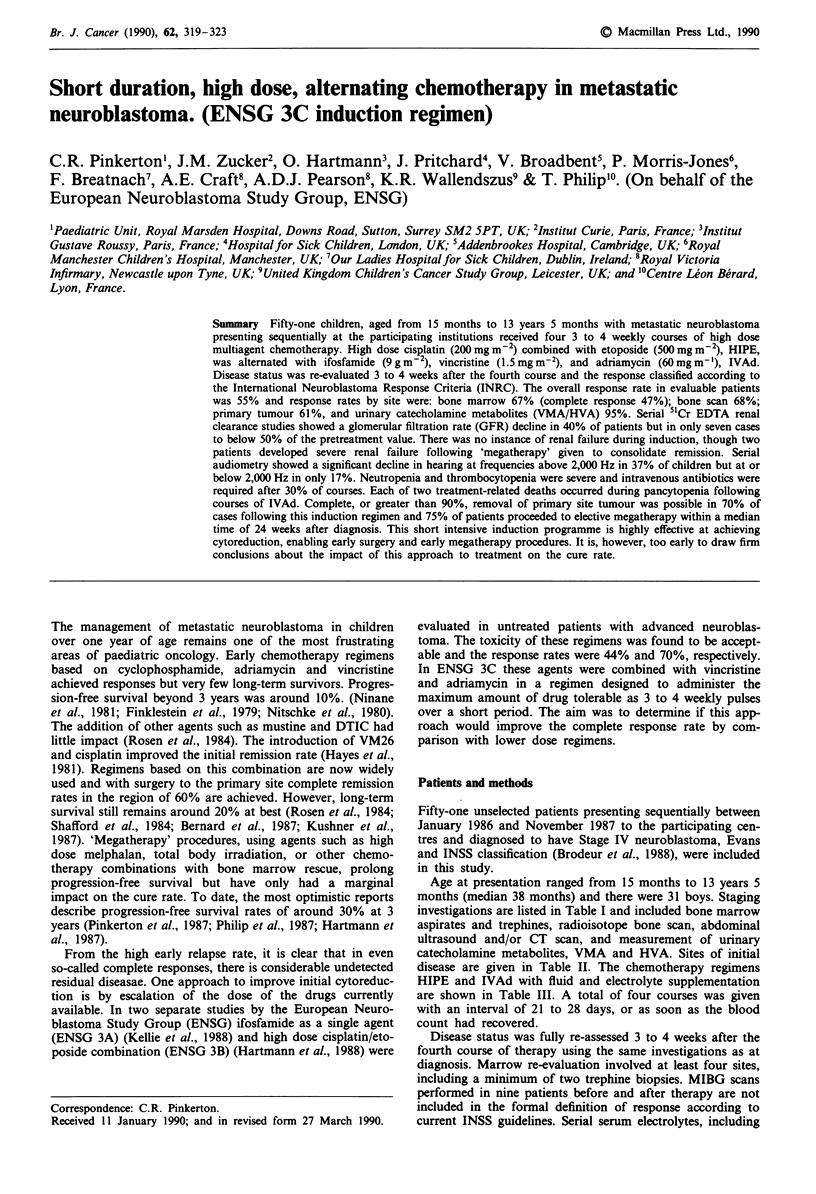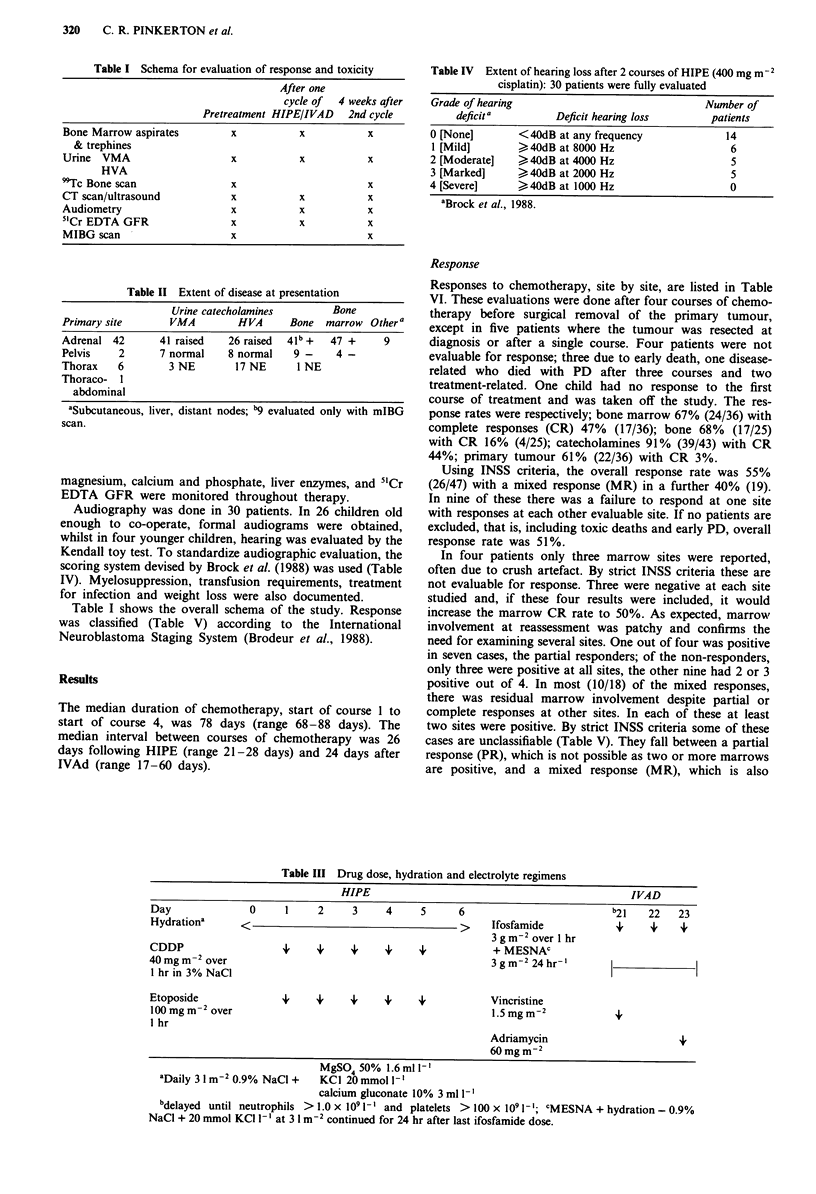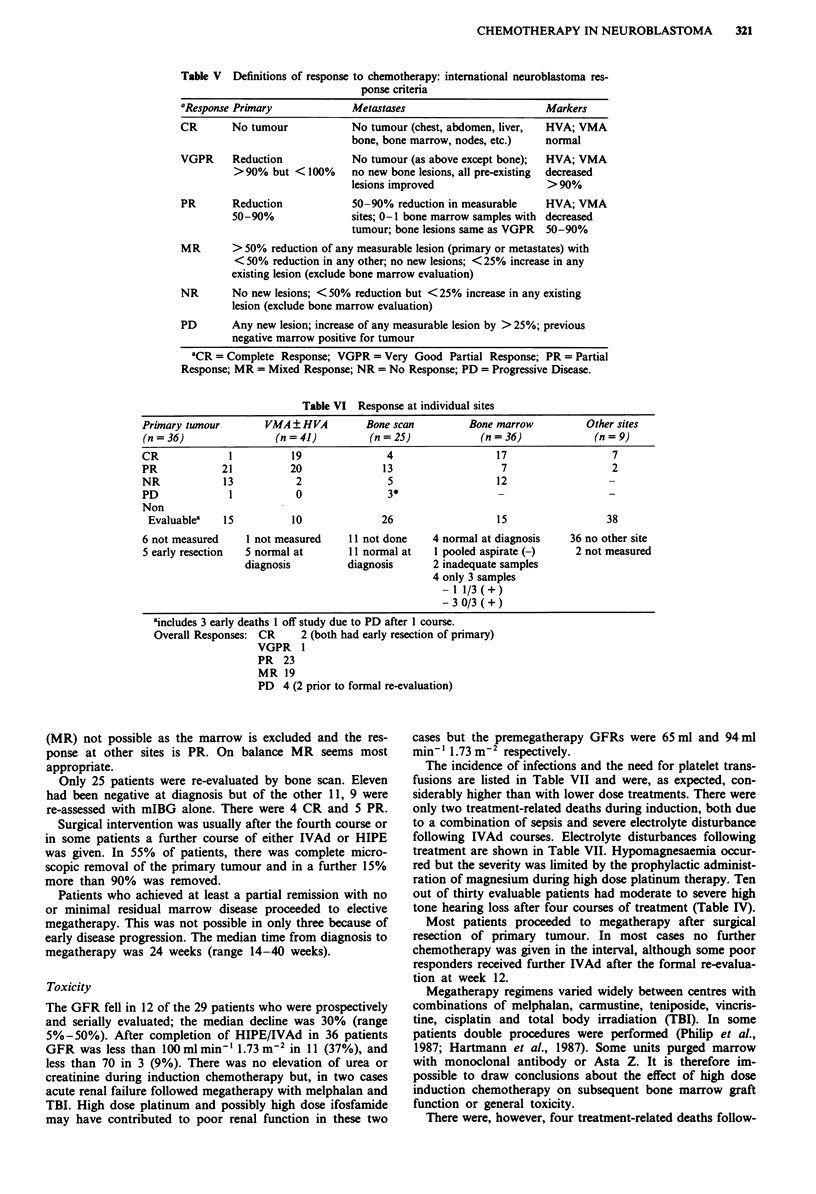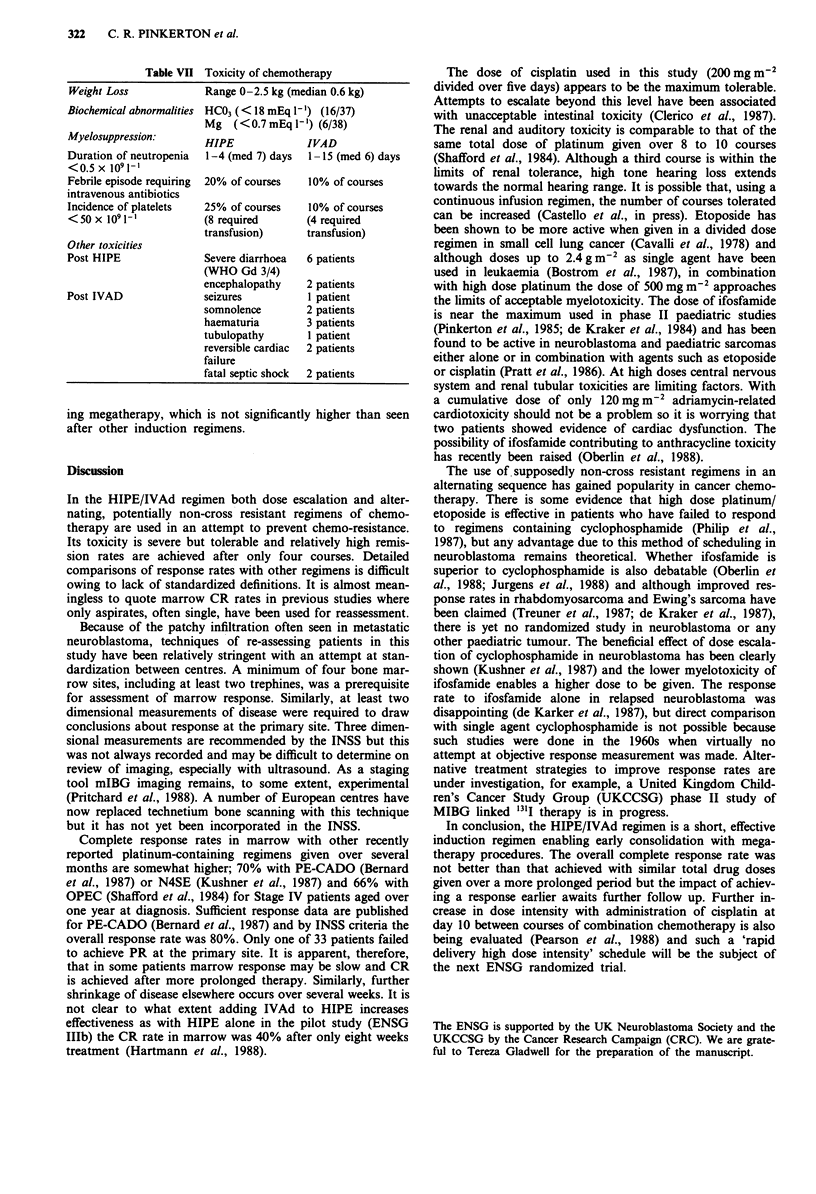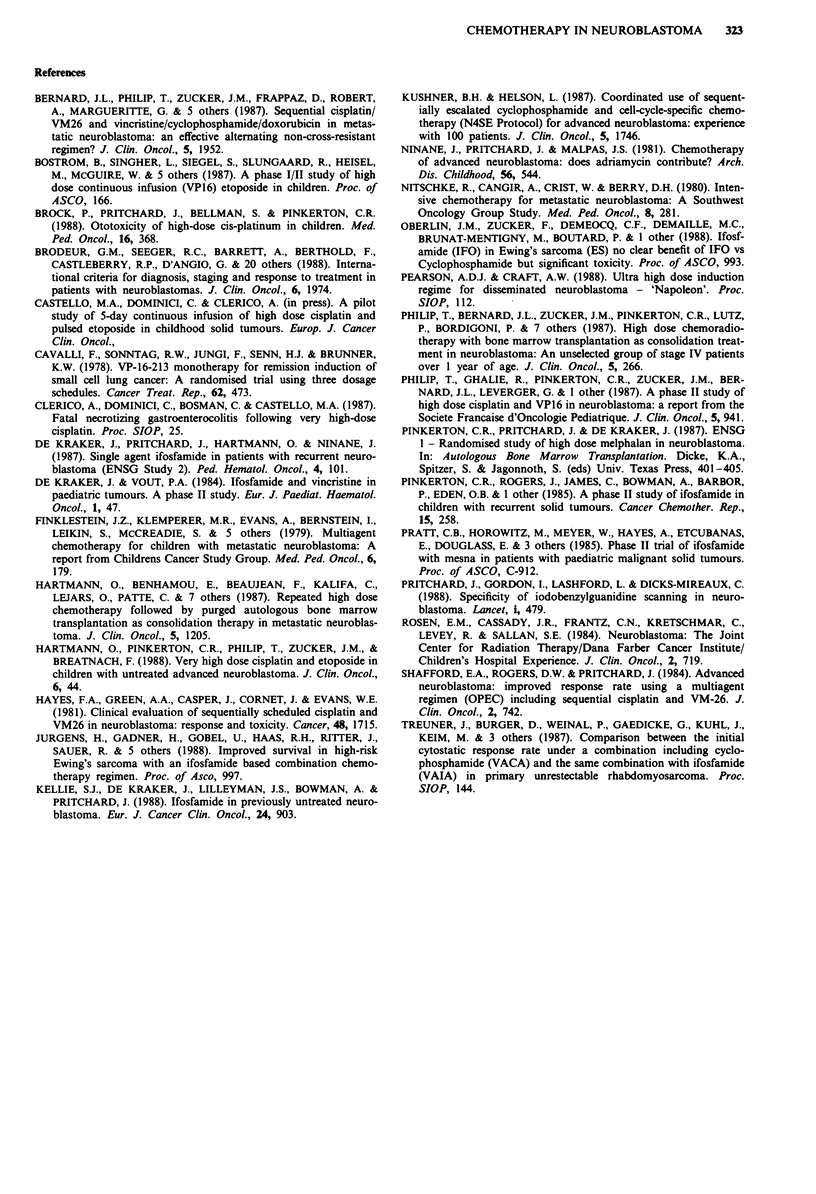# Short duration, high dose, alternating chemotherapy in metastatic neuroblastoma. (ENSG 3C induction regimen). The European Neuroblastoma Study Group.

**DOI:** 10.1038/bjc.1990.286

**Published:** 1990-08

**Authors:** C. R. Pinkerton, J. M. Zucker, O. Hartmann, J. Pritchard, V. Broadbent, P. Morris-Jones, F. Breatnach, A. E. Craft, A. D. Pearson, K. R. Wallendszus

**Affiliations:** Paediatric Unit, Royal Marsden Hospital, Sutton, Surrey, U.K.

## Abstract

Fifty-one children, aged from 15 months to 13 years 5 months with metastatic neuroblastoma presenting sequentially at the participating institutions received four 3 to 4 weekly courses of high dose multiagent chemotherapy. High dose cisplatin (200 mg m-2) combined with etoposide (500 mg m-2), HIPE, was alternated with ifosfamide (9 g m-2), vincristine (1.5 mg m-2), and adriamycin (60 mg m-1), IVAd. Disease status was re-evaluated 3 to 4 weeks after the fourth course and the response classified according to the International Neuroblastoma Response Criteria (INRC). The overall response rate in evaluable patients was 55% and response rates by site were: bone marrow 67% (complete response 47%); bone scan 68%; primary tumour 61%, and urinary catecholamine metabolites (VMA/HVA) 95%. Serial 51Cr EDTA renal clearance studies showed a glomerular filtration rate (GFR) decline in 40% of patients but in only seven cases to below 50% of the pretreatment value. There was no instance of renal failure during induction, though two patients developed severe renal failure following 'megatherapy' given to consolidate remission. Serial audiometry showed a significant decline in hearing at frequencies above 2,000 Hz in 37% of children but at or below 2,000 Hz in only 17%. Neutropenia and thrombocytopenia were severe and intravenous antibiotics were required after 30% of courses. Each of two treatment-related deaths occurred during pancytopenia following courses of IVAd. Complete, or greater than 90%, removal of primary site tumour was possible in 70% of cases following this induction regimen and 75% of patients proceeded to elective megatherapy within a median time of 24 weeks after diagnosis. This short intensive induction programme is highly effective at achieving cytoreduction, enabling early surgery and early megatherapy procedures. It is, however, too early to draw firm conclusions about the impact of this approach to treatment on the cure rate.


Br. J. Cancer (1990), 62, 319-323 © Macmillan Press Ltd., 1990

### Short duration, high dose, alternating chemotherapy in metastatic neuroblastoma. (ENSG 3C induction regimen)

C.R. Pinkerton[1], J.M. Zucker[2], O. Hartmann[3], J. Pritchard[4], V. Broadbent[5], P. Morris-Jones[6], F. Breatnach[7], A.E. Craft[8], A.D.J. Pearson[8], K.R. Wallendszus[9] & T. Philip[10]. (On behalf of the European Neuroblastoma Study Group, ENSG)

[1]*Paediatric Unit, Royal Marsden Hospital, Downs Road, Sutton, Surrey SM2 5PT, UK;* [2]*Institut Curie, Paris, France;* [3]*Institut Gustave Roussy, Paris, France;* [4]*Hospital for Sick Children, London, UK;* [5]*Addenbrookes Hospital, Cambridge, UK;* [6]*Royal Manchester Children's Hospital, Manchester, UK;* [7]*Our Ladies Hospital for Sick Children, Dublin, Ireland;* [8]*Royal Victoria Infirmary, Newcastle upon Tyne, UK;* [9]*United Kingdom Children's Cancer Study Group, Leicester, UK; and* [10]*Centre Léon Bérard, Lyon, France.*

**Summary** Fifty-one children, aged from 15 months to 13 years 5 months with metastatic neuroblastoma presenting sequentially at the participating institutions received four 3 to 4 weekly courses of high dose multiagent chemotherapy. High dose cisplatin (200 mg $m^{-2}$) combined with etoposide (500 mg $m^{-2}$), HIPE, was alternated with ifosfamide (9 g $m^{-2}$), vincristine (1.5 mg $m^{-2}$), and adriamycin (60 mg $m^{-1}$), IVAd. Disease status was re-evaluated 3 to 4 weeks after the fourth course and the response classified according to the International Neuroblastoma Response Criteria (INRC). The overall response rate in evaluable patients was 55% and response rates by site were: bone marrow 67% (complete response 47%); bone scan 68%; primary tumour 61%, and urinary catecholamine metabolites (VMA/HVA) 95%. Serial $^{51}$Cr EDTA renal clearance studies showed a glomerular filtration rate (GFR) decline in 40% of patients but in only seven cases to below 50% of the pretreatment value. There was no instance of renal failure during induction, though two patients developed severe renal failure following 'megatherapy' given to consolidate remission. Serial audiometry showed a significant decline in hearing at frequencies above 2,000 Hz in 37% of children but at or below 2,000 Hz in only 17%. Neutropenia and thrombocytopenia were severe and intravenous antibiotics were required after 30% of courses. Each of two treatment-related deaths occurred during pancytopenia following courses of IVAd. Complete, or greater than 90%, removal of primary site tumour was possible in 70% of cases following this induction regimen and 75% of patients proceeded to elective megatherapy within a median time of 24 weeks after diagnosis. This short intensive induction programme is highly effective at achieving cytoreduction, enabling early surgery and early megatherapy procedures. It is, however, too early to draw firm conclusions about the impact of this approach to treatment on the cure rate.

The management of metastatic neuroblastoma in children over one year of age remains one of the most frustrating areas of paediatric oncology. Early chemotherapy regimens based on cyclophosphamide, adriamycin and vincristine achieved responses but very few long-term survivors. Progression-free survival beyond 3 years was around 10%. (Ninane *et al.*, 1981; Finklestein *et al.*, 1979; Nitschke *et al.*, 1980). The addition of other agents such as mustine and DTIC had little impact (Rosen *et al.*, 1984). The introduction of VM26 and cisplatin improved the initial remission rate (Hayes *et al.*, 1981). Regimens based on this combination are now widely used and with surgery to the primary site complete remission rates in the region of 60% are achieved. However, long-term survival still remains around 20% at best (Rosen *et al.*, 1984; Shafford *et al.*, 1984; Bernard *et al.*, 1987; Kushner *et al.*, 1987). 'Megatherapy' procedures, using agents such as high dose melphalan, total body irradiation, or other chemotherapy combinations with bone marrow rescue, prolong progression-free survival but have only had a marginal impact on the cure rate. To date, the most optimistic reports describe progression-free survival rates of around 30% at 3 years (Pinkerton *et al.*, 1987; Philip *et al.*, 1987; Hartmann *et al.*, 1987).

From the high early relapse rate, it is clear that in even so-called complete responses, there is considerable undetected residual diseasae. One approach to improve initial cytoreduction is by escalation of the dose of the drugs currently available. In two separate studies by the European Neuroblastoma Study Group (ENSG) ifosfamide as a single agent (ENSG 3A) (Kellie *et al.*, 1988) and high dose cisplatin/etoposide combination (ENSG 3B) (Hartmann *et al.*, 1988) were evaluated in untreated patients with advanced neuroblastoma. The toxicity of these regimens was found to be acceptable and the response rates were 44% and 70%, respectively. In ENSG 3C these agents were combined with vincristine and adriamycin in a regimen designed to administer the maximum amount of drug tolerable as 3 to 4 weekly pulses over a short period. The aim was to determine if this approach would improve the complete response rate by comparison with lower dose regimens.

### Patients and methods

Fifty-one unselected patients presenting sequentially between January 1986 and November 1987 to the participating centres and diagnosed to have Stage IV neuroblastoma, Evans and INSS classification (Brodeur *et al.*, 1988), were included in this study.

Age at presentation ranged from 15 months to 13 years 5 months (median 38 months) and there were 31 boys. Staging investigations are listed in Table I and included bone marrow aspirates and trephines, radioisotope bone scan, abdominal ultrasound and/or CT scan, and measurement of urinary catecholamine metabolites, VMA and HVA. Sites of initial disease are given in Table II. The chemotherapy regimens HIPE and IVAd with fluid and electrolyte supplementation are shown in Table III. A total of four courses was given with an interval of 21 to 28 days, or as soon as the blood count had recovered.

Disease status was fully re-assessed 3 to 4 weeks after the fourth course of therapy using the same investigations as at diagnosis. Marrow re-evaluation involved at least four sites, including a minimum of two trephine biopsies. MIBG scans performed in nine patients before and after therapy are not included in the formal definition of response according to current INSS guidelines. Serial serum electrolytes, including

Correspondence: C.R. Pinkerton.
Received 11 January 1990; and in revised form 27 March 1990.

**Table I** Schema for evaluation of response and toxicity

| | Pretreatment | After one cycle of HIPE/IVAD | 4 weeks after 2nd cycle |
|---|---|---|---|
| Bone Marrow aspirates & trephines | x | x | x |
| Urine VMA HVA | x | x | x |
| $^{99}$Tc Bone scan | x | | x |
| CT scan/ultrasound | x | x | x |
| Audiometry | x | x | x |
| $^{51}$Cr EDTA GFR | x | x | x |
| MIBG scan | x | | x |

**Table II** Extent of disease at presentation

| Primary site | | Urine catecholamines VMA | HVA | Bone | Bone marrow | Other[a] |
|---|---|---|---|---|---|---|
| Adrenal | 42 | 41 raised | 26 raised | 41[b] + | 47 + | 9 |
| Pelvis | 2 | 7 normal | 8 normal | 9 − | 4 − | |
| Thorax | 6 | 3 NE | 17 NE | 1 NE | | |
| Thoraco-abdominal | 1 | | | | | |

[a]Subcutaneous, liver, distant nodes; [b]9 evaluated only with mIBG scan.

magnesium, calcium and phosphate, liver enzymes, and $^{51}$Cr EDTA GFR were monitored throughout therapy.

Audiography was done in 30 patients. In 26 children old enough to co-operate, formal audiograms were obtained, whilst in four younger children, hearing was evaluated by the Kendall toy test. To standardize audiographic evaluation, the scoring system devised by Brock *et al.* (1988) was used (Table IV). Myelosuppression, transfusion requirements, treatment for infection and weight loss were also documented.

Table I shows the overall schema of the study. Response was classified (Table V) according to the International Neuroblastoma Staging System (Brodeur *et al.*, 1988).

### Results

The median duration of chemotherapy, start of course 1 to start of course 4, was 78 days (range 68–88 days). The median interval between courses of chemotherapy was 26 days following HIPE (range 21–28 days) and 24 days after IVAd (range 17–60 days).

**Table IV** Extent of hearing loss after 2 courses of HIPE (400 mg m$^{-2}$ cisplatin): 30 patients were fully evaluated

| Grade of hearing deficit[a] | Deficit hearing loss | Number of patients |
|---|---|---|
| 0 [None] | <40dB at any frequency | 14 |
| 1 [Mild] | ≥40dB at 8000 Hz | 6 |
| 2 [Moderate] | ≥40dB at 4000 Hz | 5 |
| 3 [Marked] | ≥40dB at 2000 Hz | 5 |
| 4 [Severe] | ≥40dB at 1000 Hz | 0 |

[a]Brock *et al.*, 1988.

### *Response*

Responses to chemotherapy, site by site, are listed in Table VI. These evaluations were done after four courses of chemotherapy before surgical removal of the primary tumour, except in five patients where the tumour was resected at diagnosis or after a single course. Four patients were not evaluable for response; three due to early death, one disease-related who died with PD after three courses and two treatment-related. One child had no response to the first course of treatment and was taken off the study. The response rates were respectively; bone marrow 67% (24/36) with complete responses (CR) 47% (17/36); bone 68% (17/25) with CR 16% (4/25); catecholamines 91% (39/43) with CR 44%; primary tumour 61% (22/36) with CR 3%.

Using INSS criteria, the overall response rate was 55% (26/47) with a mixed response (MR) in a further 40% (19). In nine of these there was a failure to respond at one site with responses at each other evaluable site. If no patients are excluded, that is, including toxic deaths and early PD, overall response rate was 51%.

In four patients only three marrow sites were reported, often due to crush artefact. By strict INSS criteria these are not evaluable for response. Three were negative at each site studied and, if these four results were included, it would increase the marrow CR rate to 50%. As expected, marrow involvement at reassessment was patchy and confirms the need for examining several sites. One out of four was positive in seven cases, the partial responders; of the non-responders, only three were positive at all sites, the other nine had 2 or 3 positive out of 4. In most (10/18) of the mixed responses, there was residual marrow involvement despite partial or complete responses at other sites. In each of these at least two sites were positive. By strict INSS criteria some of these cases are unclassifiable (Table V). They fall between a partial response (PR), which is not possible as two or more marrows are positive, and a mixed response (MR), which is also

**Table III** Drug dose, hydration and electrolyte regimens

| HIPE | | | | | | | | IVAD | | | |
|---|---|---|---|---|---|---|---|---|---|---|---|
| Day | 0 | 1 | 2 | 3 | 4 | 5 | 6 | | [b]21 | 22 | 23 |
| Hydration[a] | < | — | — | — | — | — | > | Ifosfamide 3 g m$^{-2}$ over 1 hr + MESNA[c] 3 g m$^{-2}$ 24 hr$^{-1}$ | ↓ | ↓ | ↓ |
| CDDP 40 mg m$^{-2}$ over 1 hr in 3% NaCl | | ↓ | ↓ | ↓ | ↓ | ↓ | | | \|— | — | —\| |
| Etoposide 100 mg m$^{-2}$ over 1 hr | | ↓ | ↓ | ↓ | ↓ | ↓ | | Vincristine 1.5 mg m$^{-2}$ | ↓ | | |
| | | | | | | | | Adriamycin 60 mg m$^{-2}$ | | | ↓ |

[a]Daily 3 l m$^{-2}$ 0.9% NaCl + MgSO$_4$ 50% 1.6 ml l$^{-1}$, KCl 20 mmol l$^{-1}$, calcium gluconate 10% 3 ml l$^{-1}$

[b]delayed until neutrophils >1.0 × 10$^9$ l$^{-1}$ and platelets >100 × 10$^9$ l$^{-1}$; [c]MESNA + hydration – 0.9% NaCl + 20 mmol KCl l$^{-1}$ at 3 l m$^{-2}$ continued for 24 hr after last ifosfamide dose.

**Table V** Definitions of response to chemotherapy: international neuroblastoma response criteria

| [a]*Response* | *Primary* | *Metastases* | *Markers* |
|---|---|---|---|
| CR | No tumour | No tumour (chest, abdomen, liver, bone, bone marrow, nodes, etc.) | HVA; VMA normal |
| VGPR | Reduction >90% but <100% | No tumour (as above except bone); no new bone lesions, all pre-existing lesions improved | HVA; VMA decreased >90% |
| PR | Reduction 50–90% | 50–90% reduction in measurable sites; 0–1 bone marrow samples with tumour; bone lesions same as VGPR | HVA; VMA decreased 50–90% |
| MR | >50% reduction of any measurable lesion (primary or metastates) with <50% reduction in any other; no new lesions; <25% increase in any existing lesion (exclude bone marrow evaluation) | | |
| NR | No new lesions; <50% reduction but <25% increase in any existing lesion (exclude bone marrow evaluation) | | |
| PD | Any new lesion; increase of any measurable lesion by >25%; previous negative marrow positive for tumour | | |

[a]CR = Complete Response; VGPR = Very Good Partial Response; PR = Partial Response; MR = Mixed Response; NR = No Response; PD = Progressive Disease.

**Table VI** Response at individual sites

| *Primary tumour (n = 36)* | | *VMA ± HVA (n = 41)* | *Bone scan (n = 25)* | *Bone marrow (n = 36)* | *Other sites (n = 9)* |
|---|---|---|---|---|---|
| CR | 1 | 19 | 4 | 17 | 7 |
| PR | 21 | 20 | 13 | 7 | 2 |
| NR | 13 | 2 | 5 | 12 | – |
| PD | 1 | 0 | 3* | – | – |
| Non Evaluable[a] | 15 | 10 | 26 | 15 | 38 |
| 6 not measured<br>5 early resection | | 1 not measured<br>5 normal at diagnosis | 11 not done<br>11 normal at diagnosis | 4 normal at diagnosis<br>1 pooled aspirate (–)<br>2 inadequate samples<br>4 only 3 samples<br>– 1 1/3 ( + )<br>– 3 0/3 ( + ) | 36 no other site<br>2 not measured |

[a]includes 3 early deaths 1 off study due to PD after 1 course.

Overall Responses: CR 2 (both had early resection of primary)
VGPR 1
PR 23
MR 19
PD 4 (2 prior to formal re-evaluation)

(MR) not possible as the marrow is excluded and the response at other sites is PR. On balance MR seems most appropriate.

Only 25 patients were re-evaluated by bone scan. Eleven had been negative at diagnosis but of the other 11, 9 were re-assessed with mIBG alone. There were 4 CR and 5 PR.

Surgical intervention was usually after the fourth course or in some patients a further course of either IVAd or HIPE was given. In 55% of patients, there was complete microscopic removal of the primary tumour and in a further 15% more than 90% was removed.

Patients who achieved at least a partial remission with no or minimal residual marrow disease proceeded to elective megatherapy. This was not possible in only three because of early disease progression. The median time from diagnosis to megatherapy was 24 weeks (range 14–40 weeks).

### *Toxicity*

The GFR fell in 12 of the 29 patients who were prospectively and serially evaluated; the median decline was 30% (range 5%–50%). After completion of HIPE/IVAd in 36 patients GFR was less than 100 ml $min^{-1}$ 1.73 $m^{-2}$ in 11 (37%), and less than 70 in 3 (9%). There was no elevation of urea or creatinine during induction chemotherapy but, in two cases acute renal failure followed megatherapy with melphalan and TBI. High dose platinum and possibly high dose ifosfamide may have contributed to poor renal function in these two cases but the premegatherapy GFRs were 65 ml and 94 ml $min^{-1}$ 1.73 $m^{-2}$ respectively.

The incidence of infections and the need for platelet transfusions are listed in Table VII and were, as expected, considerably higher than with lower dose treatments. There were only two treatment-related deaths during induction, both due to a combination of sepsis and severe electrolyte disturbance following IVAd courses. Electrolyte disturbances following treatment are shown in Table VII. Hypomagnesaemia occurred but the severity was limited by the prophylactic administration of magnesium during high dose platinum therapy. Ten out of thirty evaluable patients had moderate to severe high tone hearing loss after four courses of treatment (Table IV).

Most patients proceeded to megatherapy after surgical resection of primary tumour. In most cases no further chemotherapy was given in the interval, although some poor responders received further IVAd after the formal re-evaluation at week 12.

Megatherapy regimens varied widely between centres with combinations of melphalan, carmustine, teniposide, vincristine, cisplatin and total body irradiation (TBI). In some patients double procedures were performed (Philip *et al.*, 1987; Hartmann *et al.*, 1987). Some units purged marrow with monoclonal antibody or Asta Z. It is therefore impossible to draw conclusions about the effect of high dose induction chemotherapy on subsequent bone marrow graft function or general toxicity.

There were, however, four treatment-related deaths follow-

**Table VII** Toxicity of chemotherapy

| | | |
|---|---|---|
| *Weight Loss* | Range 0–2.5 kg (median 0.6 kg) | |
| *Biochemical abnormalities* | $HC0_3$ (< 18 mEq $l^{-1}$) (16/37)<br>Mg (< 0.7 mEq $l^{-1}$) (6/38) | |
| *Myelosuppression:* | *HIPE* | *IVAD* |
| Duration of neutropenia < $0.5 \times 10^9 l^{-1}$ | 1–4 (med 7) days | 1–15 (med 6) days |
| Febrile episode requiring intravenous antibiotics | 20% of courses | 10% of courses |
| Incidence of platelets < $50 \times 10^9 l^{-1}$ | 25% of courses (8 required transfusion) | 10% of courses (4 required transfusion) |
| *Other toxicities* | | |
| Post HIPE | Severe diarrhoea (WHO Gd 3/4) | 6 patients |
| | encephalopathy | 2 patients |
| Post IVAD | seizures | 1 patient |
| | somnolence | 2 patients |
| | haematuria | 3 patients |
| | tubulopathy | 1 patient |
| | reversible cardiac failure | 2 patients |
| | fatal septic shock | 2 patients |

ing megatherapy, which is not significantly higher than seen after other induction regimens.

### Discussion

In the HIPE/IVAd regimen both dose escalation and alternating, potentially non-cross resistant regimens of chemotherapy are used in an attempt to prevent chemo-resistance. Its toxicity is severe but tolerable and relatively high remission rates are achieved after only four courses. Detailed comparisons of response rates with other regimens is difficult owing to lack of standardized definitions. It is almost meaningless to quote marrow CR rates in previous studies where only aspirates, often single, have been used for reassessment.

Because of the patchy infiltration often seen in metastatic neuroblastoma, techniques of re-assessing patients in this study have been relatively stringent with an attempt at standardization between centres. A minimum of four bone marrow sites, including at least two trephines, was a prerequisite for assessment of marrow response. Similarly, at least two dimensional measurements of disease were required to draw conclusions about response at the primary site. Three dimensional measurements are recommended by the INSS but this was not always recorded and may be difficult to determine on review of imaging, especially with ultrasound. As a staging tool mIBG imaging remains, to some extent, experimental (Pritchard *et al.*, 1988). A number of European centres have now replaced technetium bone scanning with this technique but it has not yet been incorporated in the INSS.

Complete response rates in marrow with other recently reported platinum-containing regimens given over several months are somewhat higher; 70% with PE-CADO (Bernard *et al.*, 1987) or N4SE (Kushner *et al.*, 1987) and 66% with OPEC (Shafford *et al.*, 1984) for Stage IV patients aged over one year at diagnosis. Sufficient response data are published for PE-CADO (Bernard *et al.*, 1987) and by INSS criteria the overall response rate was 80%. Only one of 33 patients failed to achieve PR at the primary site. It is apparent, therefore, that in some patients marrow response may be slow and CR is achieved after more prolonged therapy. Similarly, further shrinkage of disease elsewhere occurs over several weeks. It is not clear to what extent adding IVAd to HIPE increases effectiveness as with HIPE alone in the pilot study (ENSG IIIb) the CR rate in marrow was 40% after only eight weeks treatment (Hartmann *et al.*, 1988).

The dose of cisplatin used in this study (200 mg $m^{-2}$ divided over five days) appears to be the maximum tolerable. Attempts to escalate beyond this level have been associated with unacceptable intestinal toxicity (Clerico *et al.*, 1987). The renal and auditory toxicity is comparable to that of the same total dose of platinum given over 8 to 10 courses (Shafford *et al.*, 1984). Although a third course is within the limits of renal tolerance, high tone hearing loss extends towards the normal hearing range. It is possible that, using a continuous infusion regimen, the number of courses tolerated can be increased (Castello *et al.*, in press). Etoposide has been shown to be more active when given in a divided dose regimen in small cell lung cancer (Cavalli *et al.*, 1978) and although doses up to 2.4 g $m^{-2}$ as single agent have been used in leukaemia (Bostrom *et al.*, 1987), in combination with high dose platinum the dose of 500 mg $m^{-2}$ approaches the limits of acceptable myelotoxicity. The dose of ifosfamide is near the maximum used in phase II paediatric studies (Pinkerton *et al.*, 1985; de Kraker *et al.*, 1984) and has been found to be active in neuroblastoma and paediatric sarcomas either alone or in combination with agents such as etoposide or cisplatin (Pratt *et al.*, 1986). At high doses central nervous system and renal tubular toxicities are limiting factors. With a cumulative dose of only 120 mg $m^{-2}$ adriamycin-related cardiotoxicity should not be a problem so it is worrying that two patients showed evidence of cardiac dysfunction. The possibility of ifosfamide contributing to anthracycline toxicity has recently been raised (Oberlin *et al.*, 1988).

The use of supposedly non-cross resistant regimens in an alternating sequence has gained popularity in cancer chemotherapy. There is some evidence that high dose platinum/etoposide is effective in patients who have failed to respond to regimens containing cyclophosphamide (Philip *et al.*, 1987), but any advantage due to this method of scheduling in neuroblastoma remains theoretical. Whether ifosfamide is superior to cyclophosphamide is also debatable (Oberlin *et al.*, 1988; Jurgens *et al.*, 1988) and although improved response rates in rhabdomyosarcoma and Ewing's sarcoma have been claimed (Treuner *et al.*, 1987; de Kraker *et al.*, 1987), there is yet no randomized study in neuroblastoma or any other paediatric tumour. The beneficial effect of dose escalation of cyclophosphamide in neuroblastoma has been clearly shown (Kushner *et al.*, 1987) and the lower myelotoxicity of ifosfamide enables a higher dose to be given. The response rate to ifosfamide alone in relapsed neuroblastoma was disappointing (de Karker *et al.*, 1987), but direct comparison with single agent cyclophosphamide is not possible because such studies were done in the 1960s when virtually no attempt at objective response measurement was made. Alternative treatment strategies to improve response rates are under investigation, for example, a United Kingdom Children's Cancer Study Group (UKCCSG) phase II study of MIBG linked $^{131}I$ therapy is in progress.

In conclusion, the HIPE/IVAd regimen is a short, effective induction regimen enabling early consolidation with megatherapy procedures. The overall complete response rate was not better than that achieved with similar total drug doses given over a more prolonged period but the impact of achieving a response earlier awaits further follow up. Further increase in dose intensity with administration of cisplatin at day 10 between courses of combination chemotherapy is also being evaluated (Pearson *et al.*, 1988) and such a 'rapid delivery high dose intensity' schedule will be the subject of the next ENSG randomized trial.

The ENSG is supported by the UK Neuroblastoma Society and the UKCCSG by the Cancer Research Campaign (CRC). We are grateful to Tereza Gladwell for the preparation of the manuscript.

### References

BERNARD, J.L., PHILIP, T., ZUCKER, J.M., FRAPPAZ, D., ROBERT, A., MARGUERITTE, G. & 5 others (1987). Sequential cisplatin/VM26 and vincristine/cyclophosphamide/doxorubicin in metastatic neuroblastoma: an effective alternating non-cross-resistant regimen? *J. Clin. Oncol.*, **5**, 1952.

BOSTROM, B., SINGHER, L., SIEGEL, S., SLUNGAARD, R., HEISEL, M., MCGUIRE, W. & 5 others (1987). A phase I/II study of high dose continuous infusion (VP16) etoposide in children. *Proc. of ASCO*, 166.

BROCK, P., PRITCHARD, J., BELLMAN, S. & PINKERTON, C.R. (1988). Ototoxicity of high-dose cis-platinum in children. *Med. Ped. Oncol.*, **16**, 368.

BRODEUR, G.M., SEEGER, R.C., BARRETT, A., BERTHOLD, F., CASTLEBERRY, R.P., D'ANGIO, G. & 20 others (1988). International criteria for diagnosis, staging and response to treatment in patients with neuroblastomas. *J. Clin. Oncol.*, **6**, 1974.

CASTELLO, M.A., DOMINICI, C. & CLERICO, A. (in press). A pilot study of 5-day continuous infusion of high dose cisplatin and pulsed etoposide in childhood solid tumours. *Europ. J. Cancer Clin. Oncol.*,

CAVALLI, F., SONNTAG, R.W., JUNGI, F., SENN, H.J. & BRUNNER, K.W. (1978). VP-16-213 monotherapy for remission induction of small cell lung cancer: A randomised trial using three dosage schedules. *Cancer Treat. Rep.*, **62**, 473.

CLERICO, A., DOMINICI, C., BOSMAN, C. & CASTELLO, M.A. (1987). Fatal necrotizing gastroenterocolitis following very high-dose cisplatin. *Proc. SIOP*, 25.

DE KRAKER, J., PRITCHARD, J., HARTMANN, O. & NINANE, J. (1987). Single agent ifosfamide in patients with recurrent neuroblastoma (ENSG Study 2). *Ped. Hematol. Oncol.*, **4**, 101.

DE KRAKER, J. & VOUT, P.A. (1984). Ifosfamide and vincristine in paediatric tumours. A phase II study. *Eur. J. Paediat. Haematol. Oncol.*, **1**, 47.

FINKLESTEIN, J.Z., KLEMPERER, M.R., EVANS, A., BERNSTEIN, I., LEIKIN, S., MCCREADIE, S. & 5 others (1979). Multiagent chemotherapy for children with metastatic neuroblastoma: A report from Childrens Cancer Study Group. *Med. Ped. Oncol.*, **6**, 179.

HARTMANN, O., BENHAMOU, E., BEAUJEAN, F., KALIFA, C., LEJARS, O., PATTE, C. & 7 others (1987). Repeated high dose chemotherapy followed by purged autologous bone marrow transplantation as consolidation therapy in metastatic neuroblastoma. *J. Clin. Oncol.*, **5**, 1205.

HARTMANN, O., PINKERTON, C.R., PHILIP, T., ZUCKER, J.M., & BREATNACH, F. (1988). Very high dose cisplatin and etoposide in children with untreated advanced neuroblastoma. *J. Clin. Oncol.*, **6**, 44.

HAYES, F.A., GREEN, A.A., CASPER, J., CORNET, J. & EVANS, W.E. (1981). Clinical evaluation of sequentially scheduled cisplatin and VM26 in neuroblastoma: response and toxicity. *Cancer*, **48**, 1715.

JURGENS, H., GADNER, H., GOBEL, U., HAAS, R.H., RITTER, J., SAUER, R. & 5 others (1988). Improved survival in high-risk Ewing's sarcoma with an ifosfamide based combination chemotherapy regimen. *Proc. of Asco*, 997.

KELLIE, S.J., DE KRAKER, J., LILLEYMAN, J.S., BOWMAN, A. & PRITCHARD, J. (1988). Ifosfamide in previously untreated neuroblastoma. *Eur. J. Cancer Clin. Oncol.*, **24**, 903.

KUSHNER, B.H. & HELSON, L. (1987). Coordinated use of sequentially escalated cyclophosphamide and cell-cycle-specific chemotherapy (N4SE Protocol) for advanced neuroblastoma: experience with 100 patients. *J. Clin. Oncol.*, **5**, 1746.

NINANE, J., PRITCHARD, J. & MALPAS, J.S. (1981). Chemotherapy of advanced neuroblastoma: does adriamycin contribute? *Arch. Dis. Childhood*, **56**, 544.

NITSCHKE, R., CANGIR, A., CRIST, W. & BERRY, D.H. (1980). Intensive chemotherapy for metastatic neuroblastoma: A Southwest Oncology Group Study. *Med. Ped. Oncol.*, **8**, 281.

OBERLIN, J.M., ZUCKER, F., DEMEOCQ, C.F., DEMAILLE, M.C., BRUNAT-MENTIGNY, M., BOUTARD, P. & 1 other (1988). Ifosfamide (IFO) in Ewing's sarcoma (ES) no clear benefit of IFO vs Cyclophosphamide but significant toxicity. *Proc. of ASCO*, 993.

PEARSON, A.D.J. & CRAFT, A.W. (1988). Ultra high dose induction regime for disseminated neuroblastoma – 'Napoleon'. *Proc. SIOP*, 112.

PHILIP, T., BERNARD, J.L., ZUCKER, J.M., PINKERTON, C.R., LUTZ, P., BORDIGONI, P. & 7 others (1987). High dose chemoradiotherapy with bone marrow transplantation as consolidation treatment in neuroblastoma: An unselected group of stage IV patients over 1 year of age. *J. Clin. Oncol.*, **5**, 266.

PHILIP, T., GHALIE, R., PINKERTON, C.R., ZUCKER, J.M., BERNARD, J.L., LEVERGER, G. & 1 other (1987). A phase II study of high dose cisplatin and VP16 in neuroblastoma: a report from the Societe Francaise d'Oncologie Pediatrique. *J. Clin. Oncol.*, **5**, 941.

PINKERTON, C.R., PRITCHARD, J. & DE KRAKER, J. (1987). ENSG 1 – Randomised study of high dose melphalan in neuroblastoma. In: *Autologous Bone Marrow Transplantation*. Dicke, K.A., Spitzer, S. & Jagonnoth, S. (eds) Univ. Texas Press, 401–405.

PINKERTON, C.R., ROGERS, J., JAMES, C., BOWMAN, A., BARBOR, P., EDEN, O.B. & 1 other (1985). A phase II study of ifosfamide in children with recurrent solid tumours. *Cancer Chemother. Rep.*, **15**, 258.

PRATT, C.B., HOROWITZ, M., MEYER, W., HAYES, A., ETCUBANAS, E., DOUGLASS, E. & 3 others (1985). Phase II trial of ifosfamide with mesna in patients with paediatric malignant solid tumours. *Proc. of ASCO*, C-912.

PRITCHARD, J., GORDON, I., LASHFORD, L. & DICKS-MIREAUX, C. (1988). Specificity of iodobenzylguanidine scanning in neuroblastoma. *Lancet*, **i**, 479.

ROSEN, E.M., CASSADY, J.R., FRANTZ, C.N., KRETSCHMAR, C., LEVEY, R. & SALLAN, S.E. (1984). Neuroblastoma: The Joint Center for Radiation Therapy/Dana Farber Cancer Institute/Children's Hospital Experience. *J. Clin. Oncol.*, **2**, 719.

SHAFFORD, E.A., ROGERS, D.W. & PRITCHARD, J. (1984). Advanced neuroblastoma: improved response rate using a multiagent regimen (OPEC) including sequential cisplatin and VM-26. *J. Clin. Oncol.*, **2**, 742.

TREUNER, J., BURGER, D., WEINAL, P., GAEDICKE, G., KUHL, J., KEIM, M. & 3 others (1987). Comparison between the initial cytostatic response rate under a combination including cyclophosphamide (VACA) and the same combination with ifosfamide (VAIA) in primary unrestectable rhabdomyosarcoma. *Proc. SIOP*, 144.